# Lymphocyte-Rich Hepatocellular Carcinoma with Multiple Lymphadenopathy and Positive Epstein–Barr Virus Encoding Region

**DOI:** 10.1155/2023/4797233

**Published:** 2023-08-07

**Authors:** Pin-Yi Wang, Yu-Hsuan Kuo, Ming-Jen Sheu, Hsing-Tao Kuo, Wen-Ying Lee, Yu-Ting Kuo, Su-Hung Wang

**Affiliations:** ^1^Division of Hepatogastroenterology, Department of Internal Medicine, Chi Mei Medical Center, Tainan, Taiwan; ^2^Division of Hematology and Oncology, Department of Internal Medicine, Chi Mei Medical Center, Tainan, Taiwan; ^3^Department of Pathology, Chi Mei Medical Center, Tainan, Taiwan; ^4^Department of Radiology, Chi Mei Medical Center, Tainan, Taiwan

## Abstract

Lymphocyte-rich hepatocellular carcinoma (HCC) represents the rarest subtype among the various subgroups of HCC, and limited clinical data are available for this particular subtype. It is commonly observed as a solitary lesion and tends to present at an early stage. Histopathological examination typically reveals tumor cells infiltrated by a lymphocyte-rich background, leading to its designation as lymphoepithelioma-like HCC. Unlike other lymphoepithelioma-like tumors associated with the Epstein–Barr virus (EBV), lymphocyte-rich HCC is predominantly negative for EBV. This subtype is characterized by more favorable clinical outcomes and prognosis compared to conventional HCC. Here, we present a case of lymphocyte-rich hepatocellular carcinoma (HCC) characterized by the presence of bilateral hepatic tumors and concurrent multiple lymphadenopathy. Interestingly, contrary to previous literature, the examination for the Epstein–Barr virus (EBV) revealed a positive result in this particular case.

## 1. Introduction

Hepatocellular carcinoma (HCC) is a significant global health concern, ranking as the sixth most common cancer worldwide and the third leading cause of cancer-related deaths, as reported by the World Health Organization (WHO) in 2020. While the diagnosis of most HCC cases is based on characteristic imaging findings, pathological examination still plays a crucial role, particularly in cases with atypical imaging features and for providing prognostic information [1].

According to the WHO Classification of Digestive System Tumors, 5th Edition, variants of HCC can be classified into distinct subgroups based on pathological findings and molecular analyses. These subgroups include steatohepatitic, clear cell, macrotrabecular-massive, scirrhous, chromophobe, fibrolamellar, neutrophil-rich, and lymphocyte-rich subtypes [2]. Among these subtypes, lymphocyte-rich HCC is the rarest, accounting for less than 1% of HCC cases.

The molecular characteristics and pathophysiology of lymphocyte-rich HCC have not been fully elucidated to date. However, it is typically identified as a single lesion and often presents at an early stage [3]. Pathologically, lymphocyte-rich HCC is characterized by pleomorphic tumor cells infiltrated by a lymphocyte-rich background [2]. Notably, unlike other lymphoepithelioma-like tumors associated with the Epstein–Barr virus (EBV), lymphocyte-rich HCC predominantly tests negative for EBV [4]. Importantly, this subtype exhibits better clinical outcomes and prognosis compared to conventional HCC [3].

In this report, we present a case of lymphocyte-rich HCC with atypical imaging and pathology findings, highlighting the unique characteristics of this rare subtype.

## 2. Case Presentation

A 61-year-old female with occult hepatitis B infection presented with intermittent right upper quadrant abdominal dull pain lasting for 4 months. She did not exhibit jaundice, weight loss, or have a family history of malignancy. Abnormal findings on ultrasonography at a local clinic led to her transfer to a medical center hospital.

**Figure 1:**
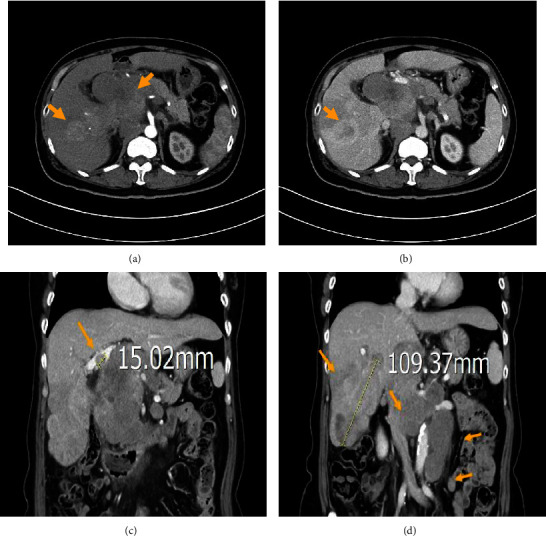
Triphasic abdominal computed tomography scan findings: The axial view revealed multiple tumor mass lesions in both the lobes, with arterial phase hyperenhancement (a) and late phase hypodensity (b). The coronal view revealed tumor thrombi in the portal vein (c) and tumor thrombi in the hepatic vein (d). The coronal view revealed maximum size of these tumor mass lesions was about 10.9 cm at S5/S6 (d). The coronal view revealed multiple enlarged lymph nodes in the paracaval and para-aortic regions of the retroperitoneum (d).

**Figure 2:**
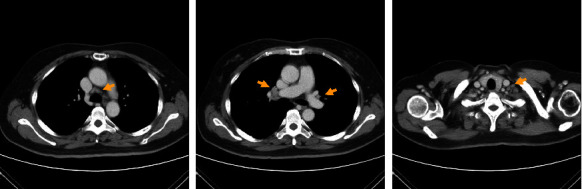
Chest computed tomography (CT) scan revealed borderline enlarged mediastinal, both hilar and left supraclavicular lymph nodes.

Repeat abdominal ultrasonography revealed the presence of multiple liver masses. Triphasic abdominal computed tomography (CT) demonstrated multiple tumor masses in both lobes of the liver, exhibiting arterial phase hyperenhancement and hypodensity in the late phase (Figures 1(a) and 1(b)). The largest tumor was measured approximately 10.9 cm (Figure 1(d)). Tumor thrombi were identified in the portal vein (Figure 1(c)) and hepatic vein (Figure 1(d)). No evidence of liver cirrhosis was observed on abdominal CT. Furthermore, multiple enlarged lymph nodes were discovered in the paracaval and paraaortic regions of the retroperitoneum (Figure 1(d)). Chest CT revealed borderline enlargement of mediastinal, hilar, and left supraclavicular lymph nodes (Figure 2).

**Figure 3:**
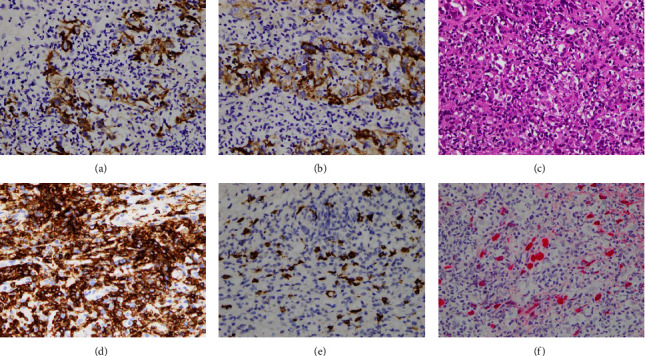
Pathological findings (original magnification, ×400): Immunohistochemical staining revealed positive AE1/AE3 (a) and positive GPC-3 (b) expression in the tumor cells. Hematoxylin and eosin staining exhibited small nests of pleomorphic tumor cells within a dense lymphoid stroma (c). The majority of lymphocytes stained positive for CD3 (d), while scattered lymphocytes showed positive staining for CD20 (e). Epstein–Barr virus encoding region in situ hybridization (EBER) demonstrated positive signals (f).

Laboratory data obtained upon admission showed the following results: aspartate aminotransferase (AST): 51 U/L, alanine aminotransferase (ALT): 29 U/L, total bilirubin: 0.38 mg/dL, international normalized ratio (INR): 1.2, HBsAg: <0.05 IU/mL, anti-HBc total: positive, and anti-HCV: negative. Tumor markers were as follows: alpha-fetoprotein (AFP): 10.3 ng/mL, carcinoembryonic antigen (CEA): 7.4 ng/mL, and carbohydrate antigen 19-9 (CA19-9): <2.0 U/mL. Liver biopsy confirmed the presence of tumor cells positive for AE1/AE3 (Figure 3(a)) and GPC-3 (Figure 3(b)) immunohistochemically. Notably, there was a dense lymphoid stroma (Figure 3(c)), and the majority of lymphocytes were positive for CD3 (Figures 3(d) and 3(e)). In addition, the tumor cells were positive for Epstein–Barr virus encoding region in situ hybridization (Figure 3(f)), indicating lymphocyte-rich hepatocellular carcinoma. Given the low incidence of HCC with multiple nonregional lymph node metastases, a percutaneous biopsy was performed on the retroperitoneal para-aortic lymph node, confirming metastatic carcinoma.

Based on the American Joint Committee on Cancer (AJCC) 8th edition and Barcelona Clinic Liver Cancer (BCLC) staging, the patient was classified as T4N1M1, stage IVB, and BCLC stage C.

The patient initially received combined target therapy and immunotherapy with bevacizumab 500 mg and atezolizumab 1200 mg once per day. However, due to immunotherapy-related neuropathy, the treatment was switched to lenvatinib 12 mg per day. After 3 months of treatment, a partial response was observed on abdominal CT. However, after 6 months, the tumor progressed again. Lenvatinib was discontinued after one year of use due to the patient's disoriented clinical condition.

## 3. Discussion

Lymphocyte-rich HCC is the rarest subtype among HCC subgroups, accounting for less than 1% of histopathological variants [2]. It has been observed to predominantly affect females based on several case reviews [3]. Approximately half of the cases have been reported to have underlying cirrhosis or chronic infection with hepatitis B or hepatitis C [3, 5]. In the present case, the patient had occult hepatitis B infection.

Lymphocyte-rich HCC is typically discovered as a single lesion and tends to present at an early stage [3]. However, in this particular case, imaging studies revealed bilateral hepatic tumors with multiple lymphadenopathy involving the retroperitoneum, mediastinum, and left supraclavicular area, indicating an advanced stage. Regional lymph nodes, especially those in the retroperitoneal and perihepatic areas, are the most common sites of spread for conventional HCC [6]. The presence of HCC with multiple lymphadenopathy in different locations is rare, suggesting the need to consider other differential diagnoses such as lymphoma or metastasis from other origins. Therefore, a lymph node biopsy was performed in this case, confirming metastatic carcinoma from HCC. It is worth noting that the combination of lymph node metastasis in both the thoracic and abdominal cavities in lymphocyte-rich HCC is a rare finding, with no reported cases documented. Our patient appears to be the first case illustrating this particular presentation. This observation raises interesting questions about the association between HCC with multiple lymph node metastases in distinct sites and lymphocyte-rich HCC. Further research is warranted to investigate this relationship.

Typical pathology findings of lymphocyte-rich HCC reveal poorly or undifferentiated tumor cells infiltrated with a lymphocyte-rich background [5]. Due to the significant intratumoral infiltration of lymphocytes, this subtype is also known as lymphoepithelioma-like HCC (LEL-HCC) [2]. The term “lymphoepithelioma” was initially used to describe tumors in the nasopharynx [3, 7]. Tumors occurring outside the nasopharynx are referred to as lymphoepithelioma-like carcinomas (LELCs) [3]. LELCs are epithelial neoplasms associated with the Epstein–Barr virus (EBV) [7]. The majority of nasopharyngeal carcinomas (NPC) are EBV-related [8], and lymphoepithelioma-like carcinomas in the lungs and stomach are also associated with EBV [7, 9]. Primary hepatic LELC includes two categories: lymphoepithelioma-like cholangiocarcinoma (LEL-CC) and lymphoepithelioma-like hepatocellular carcinoma (LEL-HCC). LEL-CC is frequently correlated with EBV [4]. However, it should be noted that LEL-HCC is mostly negative for EBV, which distinguishes it from most LELCs in other locations [3, 5].

For EBV detection, plasma EBV PCR is more accurate in determining viral load compared to Epstein–Barr virus encoding region in situ hybridization (EBER) [10]. Recent studies have shown that plasma EBV DNA analysis using real-time PCR is valuable for the early detection, prognostication, and monitoring of treatment response in NPC [10, 11]. In this case, a positive EBER result was obtained, which differs from the majority of published literature. However, serum EBV PCR data were not collected at the beginning of treatment, and further analysis was not conducted due to the progression of the patient's clinical condition and the absence of further treatment plans. It is intriguing to consider the potential use of serum EBV PCR as a tumor marker for other lymphoepithelioma-like tumors. However, since most lymphocyte-rich HCC cases are negative for EBV, it is not appropriate to utilize EBV PCR as a screening tool for lymphocyte-rich HCC. Nonetheless, EBV PCR may serve as a tool for prognostication and monitoring of treatment response in EBV-positive lymphocyte-rich HCC. Further studies are needed to confirm this hypothesis.

Immunohistochemistry analysis of lymphocyte-rich HCC demonstrates positive expression for cytokeratin and markers of hepatic differentiation, such as arginase, glypican 3, and hepatocyte paraffin 1 [12]. Tumor-infiltrating lymphocytes in lymphocyte-rich HCC mainly consist of CD3 and CD8 positive T lymphocytes, along with scattered CD20 positive B-lymphocytes [3, 13]. Recent studies have shown a correlation between lymphocyte-rich HCC and high expression of PD-L1 [14]. In this case, the typical pathology findings, including extensive intratumoral infiltration of T lymphocytes, positive cytokeratin (AE1/AE3) and hepatic differentiation markers (GPC-3), and frequent PD-L1 expression, were consistent with lymphocyte-rich HCC.

The clinical outcomes and prognosis of lymphocyte-rich HCC are generally better than those of conventional HCC, despite high PD-L1 expression. One review reported better overall survival (5-year survival: 94.1% vs. 63.9%; *P*=0.007) and progression-free survival (5-year survival: 87.8% vs. 46.6%; *P*=0.002) in lymphocyte-rich HCC compared to conventional HCC [3]. Treatment for lymphocyte-rich HCC is similar to conventional HCC, with the first-line therapy for advanced cases being a combination of targeted therapy and immunotherapy, such as bevacizumab plus atezolizumab [14, 15]. A study investigating the genomic landscape of 12 lymphocyte-rich HCC cases using whole-exome sequencing suggested that molecular alterations in lymphocyte-rich HCC may render it more susceptible to immunotherapy than conventional HCC [16]. However, research has shown that high EBV positivity in HCC with lymphocyte infiltration is associated with poorer recurrence-free survival and overall survival, indicating that EBV may not be a reliable prognostic marker in EBV-positive lymphocyte-rich HCC [17].

In this case, the patient had EBV-positive lymphocyte-rich HCC and received one course of treatment with targeted therapy combined with immunotherapy, followed by lenvatinib due to adverse effects. Although the tumor initially showed a partial response, it eventually progressed after approximately 6 months, and the treatment was discontinued after 1 year due to worsening clinical conditions.

In conclusion, this case represents the rarest subtype of HCC with multiple lymphadenopathy in different locations and positive EBER, which is not entirely consistent with most published cases. It is expected that this case will contribute to expanding our understanding of this disease.

## Data Availability

The data used to support the findings of this case report are included within the article.
